#  A close look at lay-led self-management programs for chronic diseases and health care utilisation: A systematic review and meta-analysis

**DOI:** 10.3205/000269

**Published:** 2019-04-16

**Authors:** Mareike Lederle, Eva-Maria Bitzer

**Affiliations:** 1 Pädagogische Hochschule Freiburg, Germany

## Abstract

**Introduction:** Chronically ill people are confronted with significant challenges when dealing with health care services. Lay-led self-management programs aim to improve self-management skills and might enable patients to make appropriate decisions as to when to use health care services. Contrary to the general attitude that self-management programs reduce health care utilisation, we suspect that better self-management skills lead to increased or possibly more appropriate health care utilisation. Our review and meta-analysis sheds light on the relationship between lay-led self-management programs and health care utilisation.

**Methods:** In March 2017, we searched 7 databases (CDSR, DARE, HTA, Medline, CINAHL, PsycInfo, and SSCI) to perform a systematic review and meta-analysis to identify studies that reported empirical data on lay-led self-management programs and health care utilisation. We extracted the characteristics of all primary studies and the data of four indicators of utilisation (physician visits, emergency department visits, hospital admissions, and length of stay in hospital), and analysed the role of health care utilisation in these studies. We present the results in frequency tables and as a conventional meta-analysis with the standardized mean difference (SMD), 95% confidence intervals (CI), and pooled overall effect sizes using RevMan 5.3.5. The manuscript follows the PRISMA checklist.

**Results:** Overall, we include 49 primary studies; 10 studies provided sufficient data for the meta-analysis. Health care utilisation played a different role in the studies; 15 studies reported a clear explicit hypothesis on the influence of a lay-led self-management program on health care utilisation, and 17 studies assumed an implicit assumption. 8 studies discussed the possibility that a lay-led self-management program could lead to more appropriate health care utilisation. The meta-analysis showed mixed results, and many effect sizes were not statistically significant. The participants of a lay-led self-management program had fewer emergency department visits (SMD: –0.08; 95% CI: –0.15 to –0.01; p=0.02) than the control group.

**Conclusion:** Although the statistically significant effects of the meta-analysis are low, our overall findings show that only a small number of the included studies tackled the task of comprehensively investigating self-management skills in the context of health care utilisation. This fails to do justice to the potential of self-management programs. It is essential to consider the appropriateness of health care utilisation. We propose the term *self-management-sensitive utilisation* for this purpose.

## Zusammenfassung

**Einleitung:** Chronisch Erkrankte stehen vor deutlichen Herausforderungen im Umgang mit dem Gesundheitssystem. Peergestützte Selbstmanagementprogramme können unterstützend im Umgang mit der Erkrankung wirken und Patienten befähigen, angemessene Entscheidungen zu treffen, z.B. in welchen Situationen sie das Gesundheitssystem nutzen sollten. Im Gegensatz zu der allgemeinen Einstellung, dass die Teilnahme an Selbstmanagementprogrammen die Inanspruchnahme gesundheitlicher Leistungen reduziert, vermuten wir auch die Möglichkeit einer vermehrten oder möglicherweise auch angemesseneren Inanspruchnahme gesundheitlicher Leistungen. Unsere Übersichtsarbeit und Metaanalyse soll die Beziehung von peergestützten Selbstmanagementprogrammen und der Inanspruchnahme gesundheitlicher Leistungen genauer beleuchten.

**Methodik:** Im März 2017 durchsuchten wir fünf Datenbanken (CDSR, DARE, HTA, Medline, CINAHL, PsycInfo und SSCI) im Rahmen einer systematischen Übersichtsarbeit und Metaanalyse, um Studien zu identifizieren, die Daten zur Wirksamkeit von peergestützten Selbstmanagementprogrammen auf die gesundheitliche Inanspruchnahme berichten. Wir extrahierten Merkmale aller Primärstudien und Daten zu vier Parametern der Inanspruchnahme (wie Arztbesuche, Besuche in der Notaufnahme, Krankenhausaufnahmen, Verweildauer im Krankenhaus) und analysierten die Rolle der Inanspruchnahme gesundheitlicher Leistungen in den Studien. Wir stellen die Ergebnisse in Häufigkeitstabellen sowie konventionellen Metaanalysen mit standardisierten Mittelwertsdifferenzen, 95% Konfidenzintervallen und gepoolten Gesamteffekten mit Hilfe von RevMan 5.3.5. dar. Das Manuskript orientiert sich an PRISMA.

**Ergebnisse:** Insgesamt schließen wir 49 Primärstudien ein, 10 Studien liefern ausreichende Daten für die Metaanalyse. Die Inanspruchnahme gesundheitlicher Leistungen spielt eine unterschiedliche Rolle in den Studien. 15 Studien formulieren eine explizite Hypothese zum Einfluss von peergestützten Selbstmanagementprogrammen auf die Inanspruchnahme, 17 Studien treffen eine implizite Annahme. Die Möglichkeit, dass die Teilnahme an einem peergestützten Selbstmanagementprogramm zu einer angemesseneren Inanspruchnahme gesundheitlicher Leistungen führen kann, diskutieren 8 Studien. Die Metaanalyse zeigt heterogene Ergebnisse und viele Effektgrößen sind nicht statistisch signifikant. Teilnehmer eines peergestützten Selbstmanagementprogramms suchen weniger die Notaufnahme auf (SMD –0,08, 95% CI –0,15, –0,01, p=0,02) als die Kontrollgruppe.

**Schlussfolgerung:** Obwohl die Metaanalyse wenig statistisch signifikante Ergebnisse liefert, zeigen unserer Ergebnisse insgesamt, dass nur wenige der eingeschlossenen Studien sich der Aufgabe einer komplexen Betrachtung von Selbstmanagement und der Inanspruchnahme gesundheitlicher Leistungen stellen. Dies wird dem Potenzial von Selbstmanagementprogrammen jedoch nicht gerecht. Berücksichtigt werden muss die Angemessenheit der Inanspruchnahme, wir schlagen dafür den Terminus *Selbstmanagement-sensitive Inanspruchnahme* vor.

## Introduction

Chronically ill people are confronted with significant challenges in dealing with health care services and communicating with health professionals [1], [2]. For this reason, the delivery of health care to patients with chronic illnesses requires well-structured health care providers, in addition to informed, active, and self-responsible patients [3]. Patients should be able to identify when professional help is necessary and when to seek advice from health professionals [4]. Good self-management skills might enable patients to make those decisions and to appropriately utilise the health care system [3]. Various self-management programs have been developed to promote such self-management skills. These can include generic or disease-specific interventions, and they are carried out by health professionals or, in particular, by individuals who are themselves directly or indirectly affected by a chronic condition, such as the lay-led Chronic Disease Self-Management Program (CDSMP) [5], [6]. The focus of self-management programs is on promoting skills that facilitate dealing with a disease in everyday life and to help manage the associated challenges [2]. In addition, health care utilisation support is also one of the purposes of a self-management program [2], [3].

Appropriate health care utilisation consists of an interaction of different factors and stakeholders, and it may be associated with both a reduction in its overuse, as well as in improvements in its underuse and inadequate use [4]. The most widely accepted theoretical behavioural model of health care utilisation is proposed by Andersen’s Behavioural Model of Health Services Use [7]. Use is defined by determinants on an individual level and a contextual level. These include: a) predisposing factors, which depict the indirect impact of demographic characteristics, social structure, and health beliefs; b) need factors, as a direct influence of the need; as well as c) enabling factors, defined as prerequisites for health care utilisation, such as health insurance and accessibility.

In this model, the promotion of self-management begins with need factors, thereby theoretically affecting health care utilisation.

There is a strong expectation among professionals that health care utilisation will be reduced through a self-management program [8], [9]. For example, physicians measure the success of self-management by the reduction in patients’ needs for health services and unscheduled visits [8]; in the case of previous overuse, this might be interpreted as appropriate health care utilisation. However, in cases of previous underuse, a lay-led self-management program might increase utilisation, e.g. if patients’ self-efficacy increased by participating in a lay-led self-management program and they seek further advice from a health professional. In line with this presumption are results for adolescents and young adults with different chronic diseases [10]. As reported by Gately et al. [11], we suspect the association between lay-led self-management programs and health care utilisation to be more complex. A more complex consideration might include an increase in health care utilisation in the case of underuse, and a decrease in the case of overuse; therefore, there should be evidence of health care utilisation that is both needs-based and appropriate.

In the presented review and meta-analysis, we would thus like to shed more light on the relationship between lay-led self-management programs and health care utilisation. The research questions underlying this review are:

- How is health care utilisation accounted for in the studies?
- Which explicit and implicit hypotheses do the researchers make of the effect of lay-led self-management programs on health care utilisation?
- What effects does a lay-led self-management program have on health care utilisation?
- Does participation in a self-management program lead to a more appropriate use of health services?

## Methods

The protocol of this review is registered in the PROSPERO database (CRD42017067956). We performed a meta-analysis of studies with continuous outcomes, and we also performed a qualitative synthesis given the sufficient number of studies with comparable outcomes. Compared to the protocol, we cannot comment on the effects of different chronic diseases with the available data.

### Data sources

A systematic literature search was conducted in March 2017, using the Cochrane Database of Systematic Reviews (CDSR), the Database of Abstracts of Reviews of Effects (DARE), the Health Technology Assessment Database (HTA), Medline via PubMed, CINAHL and PsycInfo via EBSCOhost, as well as the Social Science Citation Index via Web of Science. We searched and extracted primary studies from existing meta-analyses, systematic reviews, reviews, and health technology assessments conducted between 2006 and 2017. In addition, we updated the search to identify existing primary studies, from the date of the latest review search (2013–2017). The additional data were added into the syntheses as appropriate. For the search strategy, we focused on available reviews with similar topics [12], [13]. We used a combination of tags and keywords, such as, for example, ‘self-management’, ‘self-care’, ‘peer*’, ‘lay-led’, ‘chronic disease’, ‘long-term disease’, and ‘health care use’. We adapted the search strategies to the respective database.

### Study selection

All titles and abstracts of the identified results were independently examined for their relevance by 2 people (ML, SS); discrepancies between the reviewers were discussed and a consensus was reached. The matches identified in the title or abstract had to reveal that the quantitative or qualitative efficacy data with respect to a change in health care utilisation (visits to the physician, visits to the emergency department, hospital admissions, and length of stay in the hospital) were associated with lay-led self-management programs for people with chronic diseases, as this was an integral component of the publication.

The target population consisted of adults suffering from 1 or several chronic diseases, such as heart disease, type 1 and 2 diabetes, asthma, chronic obstructive pulmonary disease, arthritis, or chronic pain. We compared lay-led self-management programs, defined as a structured program for individuals with chronic diseases administered by trained affected persons who are helping the patients to improve their own health, with the standard of care. We accounted for generic and illness-specific interventions. Insofar as the publication included an interactive component between the participant and the trainer, it was not only possible to conduct the intervention in person, but also by telephone or via the Internet. Furthermore, we included primary studies comprising both peer- and expert-based perspectives, particularly if it was possible to assess the effects of the lay-led interventions separately. We excluded self-management interventions developed exclusively for children and adolescents.

### Data extraction

The characteristics of the primary studies, such as the sample size, follow-up, nature of the self-management program, examined diseases, and the outcomes (mean change value, standard deviation, n) were extracted by one person (ML) using different data-extraction tables. The methodological quality of the reviews was rated in consideration of the AMSTAR grading criteria [14], the methodological quality of randomized controlled trials (RCTs) was determined by means of the Cochrane Risk-of-Bias-Tool [15], and uncontrolled studies were assessed by means of the Quality Assessment Tool for Before-After (Pre-Post) Studies With No Control Group [16].

To analyse the role of health care utilisation in the studies, we additionally categorized the included studies in the context of a qualitative synthesis with the following keywords: ‘explicit and implicit hypothesis’, ‘direction of the formulated hypotheses’, and ‘appropriateness of health care utilisation’. ‘Explicit’ refers to the concrete description of a hypothesis, e.g., “we hypothesized that...”. As an ‘implicit’ assumption, we categorized passages if, e.g., the table shows “downwards arrow means lower score indicates better results” or if, as e.g. in the introduction, only one direction of the impact of a self-management program is addressed.

### Data analyses

We performed a meta-analysis using Review Manager 5.3.5 [17]. Based on the studies, we expect heterogeneity between studies. Therefore, we used a random-effects model and adopted a more conservative approach [18]. We tested statistical heterogeneity using a visual inspection of a forest plot, as well as via chi-squared and I^2^ statistics, labelling levels of heterogeneity as ‘low’ (0%–25%), ‘moderate’ (26%–74%), and ‘high’ (>75%) [19]. For continuous outcomes, we calculated standardized mean difference (SMD) along with their 95% confidence intervals and pooled overall effect sizes. If no mean change values or standard deviations were given, these were calculated if possible, e.g., by using confidence intervals. For each outcome, we conducted separate meta-analyses of the effects of self-management programs. If multiple outcomes existed for one parameter, we used the outcome that was most comparable to the other outcomes. The number of studies analysed depended on the number of studies reporting that outcome. Subgroup analyses were defined a priori and were performed according to the type of self-management program (generic or disease specific).

In all, this manuscript follows the PRISMA checklist, the reporting standard for systematic reviews [20].

## Results

**Figure 1:**
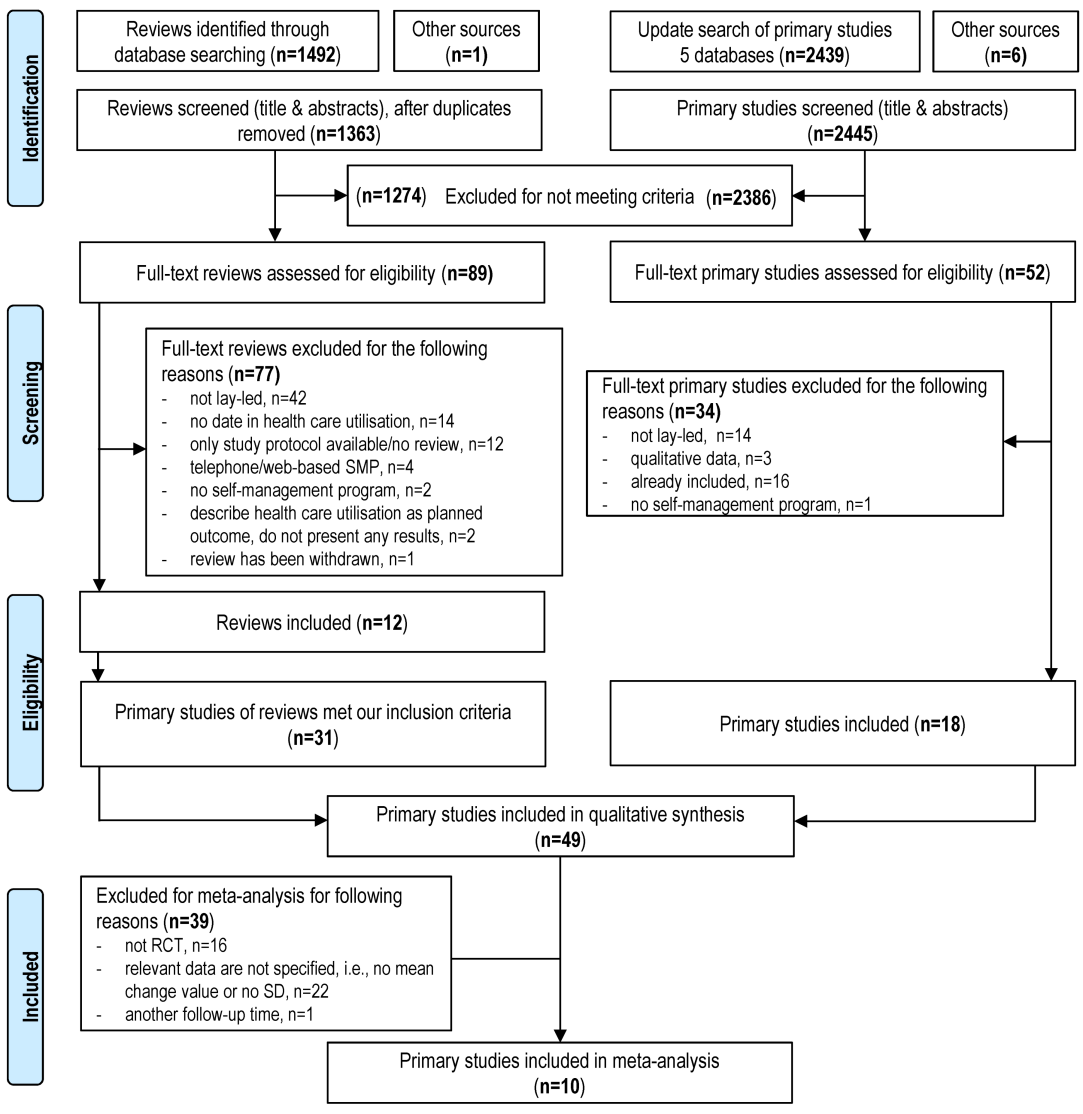
PRISMA flow diagram

We identified a total of 1,363 reviews using the initial search strategy; 2,445 references were identified from the updated search (see Figure 1 (Fig. 1)). From these two searches, we included 12 reviews, among which were 4 meta-analyses [21], [22], [23], [24], 1 Cochrane review [12], 5 systematic reviews [25], [26], [27], [28], [29], 1 narrative review [30], and 1 Health Technology Assessment [31]. The reviews were published between 2006 and 2014, the majority in 2013 and 2014, and they originated from Canada [26], [28], [31], Great Britain [12], [22], [24], [27], the USA [21], [25], and the Netherlands [29], [30]. 77 full texts were excluded for various reasons (see Figure 1 (Fig. 1)). After applying the inclusion and exclusion criteria, 31 primary studies were chronicled in the identified reviews, and 18 primary studies were included in the updated search. Thus, this review includes a total of 49 studies in 55 publications, 33 randomized controlled trials (RCTs) (67%), and 16 studies (16%) with a one-group pretest-posttest design.

The primary studies were published between 1982 and 2017 and included a total of 19,762 patients. 24 studies (49%) were conducted in the USA, 8 (16%) in Great Britain, and the remainder in Canada, the Netherlands, Australia, China, Austria, and Spain. In 28 studies (57%), the intervention consisted of the *Chronic Disease Self-Management Program*, or a modified version thereof, while the other studies investigated illness-specific procedures or other programs such as *Peer* *Support* programs. In most of the studies, health care utilisation was documented by means of self-reported answers in a questionnaire. The study follow-up ranged between 1.5 months and 2 years, with a follow-up of 4–6 months considered in the majority of the studies (84%). Patients with lung diseases, such as asthma, chronic bronchitis, emphysema or chronic obstructive pulmonary disease (53%), heart diseases (such as coronary heart disease, cardiac insufficiency, or hypertension) (41%), arthritis (43%), and diabetes (39%) were most frequently enrolled (see Table 1 (Tab. 1); for detailed characteristics of the studies, see Appendix Table 1 ).

### Quality of the examined studies

The methods detailing how subjects were allocated to the groups could only be determined in 15 of the RCTs [32], [33], [34], [35], [36], [37], [38], [39], [40], [41], [42], [43], [44], [45], [46]. Adequate procedures for secret group allocation were undertaken in 9 studies [32], [33], [34], [38], [39], [40] ,[44], [47], [48], and these included central allocation by external parties or the use of opaque, sealed envelopes. To avoid performance bias, either the study staff or the subject was blinded in only 4 studies [38], [49], [50], [51], and knowledge about allocation was not adequately prevented in any of the studies. Blinding of outcome assessors was only reported in 1 study [51]. To reduce the risk of incomplete data, missing values were imputed in 2 studies [33], [34] and an intention-to-treat analysis was conducted in 8 studies [34], [35], [38], [41], [43], [44], [48], [52]. Overall, many aspects were not described in the studies, and the risk of bias could not always be adequately assessed.

Regarding the 18 examined uncontrolled studies, there was risk of an attrition bias due to incomplete data. The follow-up rates were low, though missing values were replaced in the analysis in 3 studies [53], [54], [55]. Specific details about the sample size calculation were only provided in 1 study [56].

### Relationship between lay-led self-management programs and health care utilisation

In the following section, we describe the results while considering the role of health care utilisation in the 49 primary studies. Health care utilisation was conceptualised differently in the studies (Table 2 (Tab. 2)).

Most frequently, physicians’ visits were taken into account as an outcome parameter (92%), followed by emergency department visits (59%), length of stay in hospital (59%), and hospital admission (43%). The outcome parameters that were considered in only a few studies are summarized under ‘others’; these included physiotherapist, psychologist, alternative practitioner, nurse, or pharmacy visits. On average, 3 outcome parameters were considered in the primary studies (with a range between 1 and 11 outcomes).

#### Hypothesis

15 studies (31%) reported a clear hypothesis on the influence of a lay-led self-management program on health care utilisation. Another 17 studies (35%) did not explicitly state a hypothesis, but we can assume an implicit assumption. There were also studies in which the outcomes associated with health care utilisation were analysed, but no explicit or implicit assumptions regarding health care utilisation were mentioned. For most of the studies mentioning an explicit or implicit assumption, almost all (explicit, 87%; implicit, 100%) suspect a decrease in utilisation. Two studies expected appropriate health care utilisation [57] or an increase in the use of routine health care services and screenings [46] after participating in the lay-led self-management program.

#### Appropriate health care utilisation

With respect to their findings, 8 studies (16%) discussed the possibility that a lay-led self-management program could lead to more appropriate health care utilisation [32], [34], [39], [43], [46], [51], [53]. For example, the fact that the participants visited their physician more often: “…may be because the intervention encouraged participants to seek advice from their general practitioner” [34]. 4 studies suggested that appropriate health care utilisation is possibly promoted by a lay-led intervention [39], [43], [51], [53]; for example: “It appears that the mentees learned to seek care for symptoms earlier than they might have. The question of whether this early accessing of treatment was appropriate cannot be answered in this study […]. It also may be that patients were seeking care inappropriately” [43].

### Effects of lay-led self-management programs on health care utilisation

In the meta-analysis, we took a total of 10 primary studies into account [33], [36], [41], [46], [50], [51], [58], [59], [60], [61]. The other 39 studies were excluded from the meta-analysis for various reasons (see Figure 1 (Fig. 1)). The individual forest plots can be found in the Appendix (see Attachment 1). The number of physician visits was examined as an outcome in all 10 studies, while the number of emergency department visits was explored in 5 studies, the number of hospital admissions was investigated in 2 studies, and the length of stay in hospital was assessed in 5 studies.

Table 3 (Tab. 3) describes the effects of lay-led self-management programs on health care utilisation, as ordered by outcome parameters and type of intervention. The meta-analysis showed mixed results, and many of the overall effect sizes were not statistically significant. Statistically significant effects were only seen in decreases of health care utilisation. The participants of a lay-led self-management program had fewer emergency department visits (SMD: –0.08; 95% CI: –0.15 to –0.01; p=0.02) than the control group. There are very small trends towards increases and decreases in health care utilisation in the different outcome parameters. The subgroup analyses indicate that participants in a generic self-management program may be less likely to visit different health care providers than patients who did not participate (not significant).

## Discussion

The purposes of this study were:

1. to understand the role of health care utilisation in studies that evaluated lay-led self-management programs in chronic diseases;
2. to determine the associated hypotheses;
3. to examine the effects of lay-led self-management programs on the associated changes in health care utilisation;
4. to revise our hypothesis that better self-management skills will lead to more appropriate health care utilisation.

Although all included studies considered health care utilisation as an outcome, utilisation plays a very different role. Taking into account the potential of self-management programs to alter health care utilisation [2], [3], we did not expect such a variety of outcome indicators in the primary studies. The studies included a different number of outcomes (range: 1–11 outcomes) and considered different types of health care utilisation (for example, alternative practitioners or specialists). Consistent with several studies, physician visits, emergency department visits, length of stay in hospital, and hospital admission were considered. Almost all studies suspected a decrease in utilisation. Self-management within the Chronic Care Model aims to empower patients in decision-making processes [1], which does not exclusively mean lower health care utilisation and an associated decrease in costs. In fact, this should encompass needs-based and appropriate health care utilisation. The appropriateness of health care utilisation after a lay-led self-management program is addressed in only very few studies, and mainly appears in the discussion of the results, with the exception of one study [46] that directly hypothesized in its introduction that patients are encouraged to go to the physician more often, as based on a disease-specific intervention. In addition, when compared to other general lay-led self-management programs, this intervention was a peer health navigator intervention that was more intensive and specially designed for appropriate health care utilisation (The Bridge) [46]. It might be advisable to include similar elements in the basic structure of lay-led self-management programs to achieve greater effects on health care utilisation.

In line with other reviews [62], [63], [64], our meta-analysis showed a small impact of lay-led self-management programs on health care utilisation. We only saw reductions in emergency department visits; and effects on other indicators of health care utilisation were not apparent. It can be assumed that this decrease in health care utilisation suggests a reduction in unscheduled visits, as self-management is beneficial for reducing avoidable emergency department visits and unplanned hospital admissions [65]. However, whether this can be interpreted as appropriate cannot be answered with certainty. Theoretically, the peer support model is designed such that like-minded people help each other navigate a wide range of health services [2]. As based on the available data, it is difficult to derive findings regarding the appropriate level of health care utilisation. Part of these difficulties may lie in the fact that an exact comparison of the included studies is often impossible given the different hypotheses associated with – and the different roles played by – health care utilisation.

Hopkins pointed out as early as in 1993 that with appropriate health care utilisation, the responsibility to seek such services also rests with the patients. Enabling patients to accept responsibility and motivating them to participate in their care (empowerment) is one thing that providers can do to boost the appropriateness of health care utilisation, aside from, e.g., establishing guideline recommendations [66]; in this way, self-management programs can theoretically make a contribution to resource access. No necessary services should be withheld, but rather they should promote a critical use of health services (i.e., health literacy) and foster responsible handling of the disease [67]. A qualitative study investigating patients’ views of their disease, as well as the self-care they engage in within the context of their chronic disease and health care utilisation, likewise illustrates that a self-management program can enable patients to become more confident when managing their diseases and navigating the medical system. In this way, there may be a reduction in health care access and, when necessary, patients can increase their health care utilisation if their condition worsens, for instance [11]. 

There are various interventions that can be used to promote the appropriate use of health care services [68], [69], although an even greater focus on individual skills may be needed. To achieve effective self-management, patients need a high degree of health literacy and a strong sense of empowerment. If patients have both, they “become effective self-managers of their health using healthcare resources appropriately to optimize their health outcomes” [70]. In other words, improved patient self-management skills can make health care utilisation more appropriate. ‘Appropriate’ in this context also means that the health care system is possibly used more often after a patient participates in a lay-led self-management program; however, in that case, it should be attended at the right time and with the appropriate provider.

In Andersen’s Health Behavior Model, utilization is defined by need, predisposing, and enabling factors [7]. Self-management programs aim to improve the need factors in the Andersen model, which reflect disease characteristics. For example, self-reported health status or health related quality of life can be improved by a lay-led self-management [31], and in turn affect physician visits [71]. An appropriate health care utilisation might be a utilisation which is not accompanied by a reduction in quality of life. It can be assumed that there will be a change in health care utilisation in services that are not necessarily needed (overuse), such as the reduction of emergency department visits in our data, and an increase in the use of underused services (underuse). We propose the term *self-management-sensitive utilisation* for this purpose. Better self-management skills can theoretically lead to appropriate health care utilisation [70], but self-management certainly cannot influence all health service utilisation, since it is also influenced by numerous other factors, such as demographic characteristics (predisposing characteristics) or health service accessibility (enabling resources) [7].

When interpreting the results, it should be considered that searching for self-management programs can be very complex, as there is no unified definition and a variety of different programs. Therefore, we have based our search strategy on existing reviews and meta-analyses. Also, the quality of this review is based on the quality of the information contained in the included primary studies. It needs to be considered that due to the insufficient quality of reported data, we can only include part of the primary studies in the meta-analysis. We follow the statement of Cuijpers et al. [72] and excluded pre-post studies as well as studies with low reporting quality from the meta-analysis. However, substantial results should not be lost, so we considered all primary studies in the qualitative synthesis. In this review, only the results on health care utilisation were accounted for; other outcomes, such as clinical parameters, may also be relevant for other hypotheses and other perspectives for the effectiveness of self-management programs. Qualitative studies with a focus on the topic of appropriate health care utilisation are needed. Presumably, appropriateness cannot simply be measured in terms of frequencies in quantitative studies, but they should be investigated especially in a qualitative research design. It might be important to understand how patients’ health care utilisation changes after a lay-led self-management program and how patients go through this change. In a participatory approach it should be examined how the appropriateness of health care utilisation is understood by patients and experts. A theoretical foundation for the appropriate health care utilisation is necessary. It should take into account the responsibilities of the health care system (e.g. people-centred health care [73]), as well as opportunities where the patient takes over.

Further research is necessary to consider the impact of lay-led self-management programs on health care utilisation. In particular, high reporting quality studies with a longer follow-up are needed to better reflect the appropriateness of health care utilisation and the long-term nature of chronic diseases.

## Conclusions

Patients should be sensitized to actively participate in their own care, to better manage their illness, and to navigate through the health care system. The close association between self-management skills, health literacy, and empowerment requires further public health strategies aimed at boosting these skills of patients, thereby rendering health care utilisation more appropriate. The appropriateness of health care utilisation should be examined more thoroughly in future studies; we propose the term *self-management-sensitive utilisation* for this purpose.

## Notes

### Competing interests

The authors declare that they have no competing interests.

### Acknowledgements

We thank Sandra Stiegeler for her support in the study selection. This manuscript is supported by the cooperative doctoral study course “Health Services Research: Collaborative Care” located in Freiburg, Germany. The doctoral study course in turn is brought forward by the Ministry of Science, Research and the Arts Baden-Württemberg.

## Supplementary Material

Appendix

## Figures and Tables

**Table 1:**
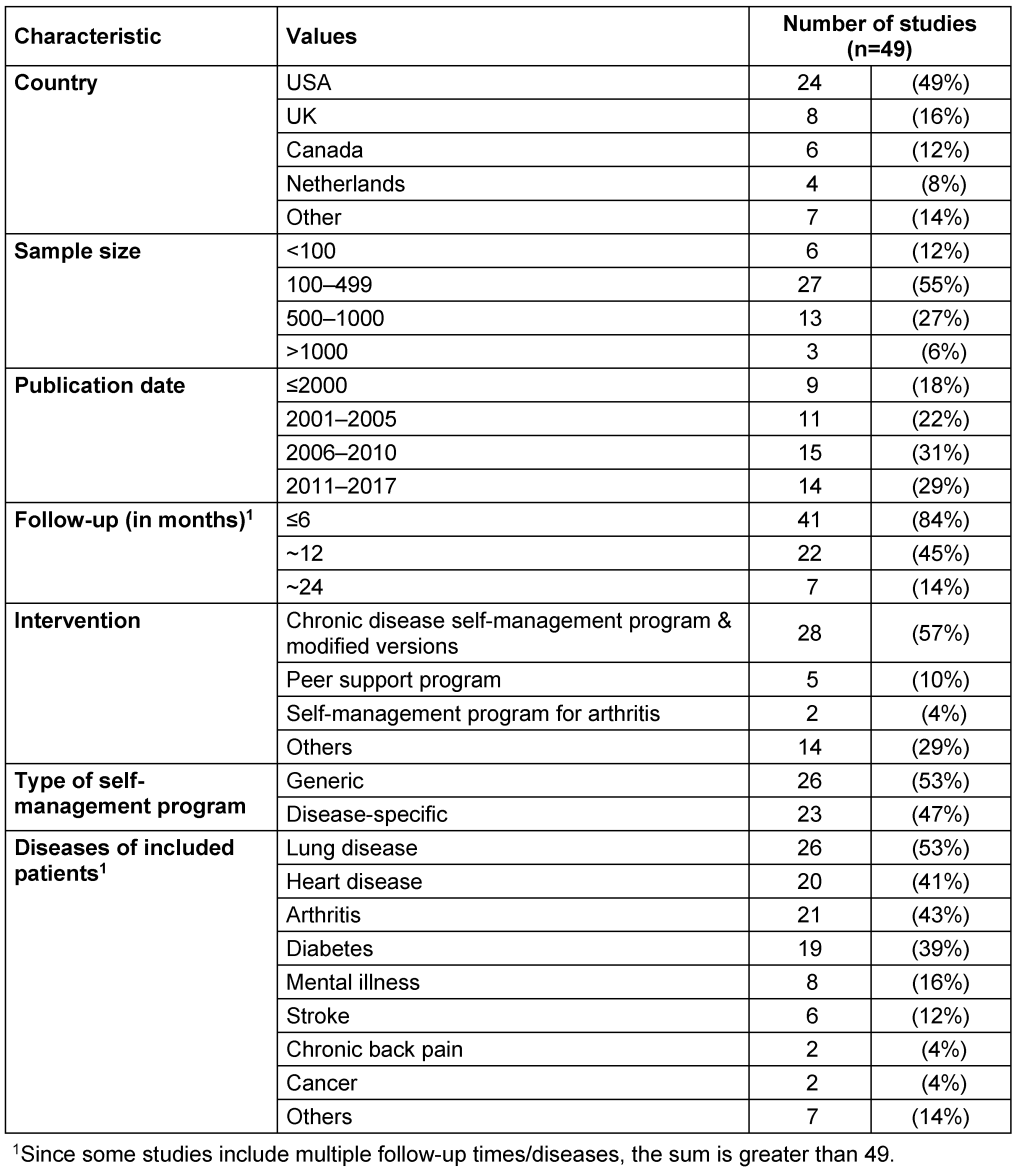
Study characteristics of the 49 primary studies

**Table 2:**
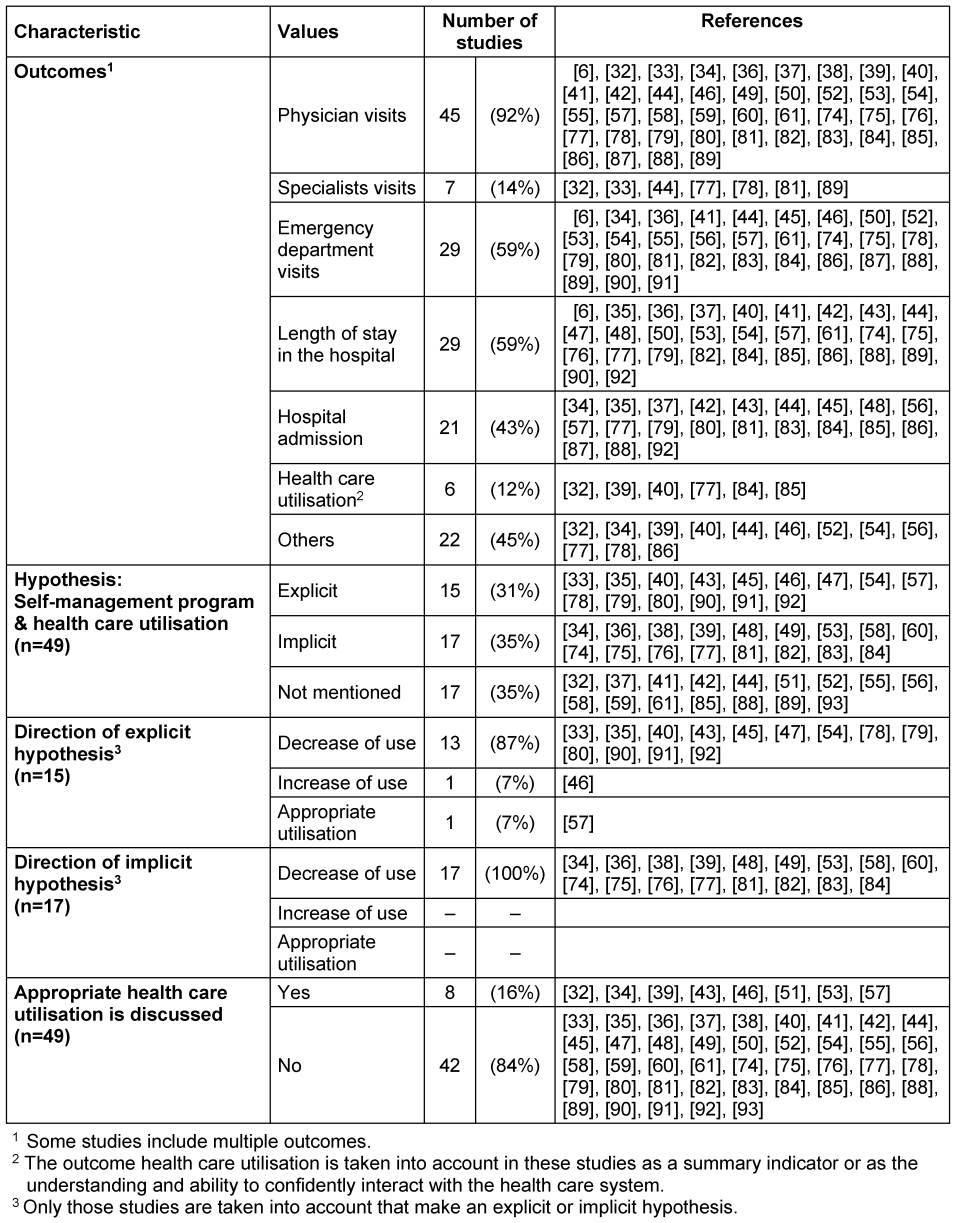
Relationship between lay-led self-management programs and health care utilisation

**Table 3:**
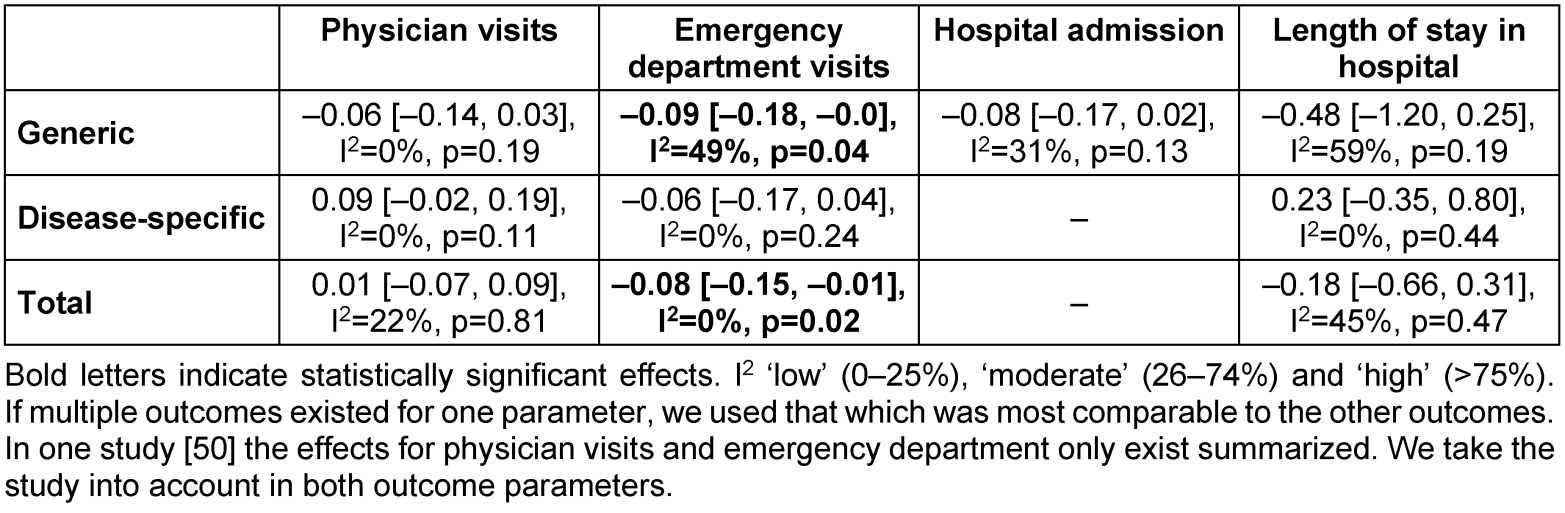
Summary – Health care utilisation (overall ES, 95% CI, I^2^, p-value)
