# Supplementary material for: A close look at lay-led self-management programs for chronic diseases and health care utilisation: A systematic review and meta-analysis
Source: Ger Med Sci. 2019 Apr 16;17:Doc03. doi: 10.3205/000269 (PMC6533545; doi:10.3205/000269)
Supplement: Appendix [file GMS-17-03-s-001.pdf]

# Appendix

## Appendix Figure 1: Number of physician visits, short follow-up

| | lay-led SMP | | | standard care | | | | Std. Mean Difference | |
|---|---|---|---|---|---|---|---|---|---|
| Study or Subgroup | Mean | SD | Total | Mean | SD | Total | Weight | IV, Random, 95% CI | Year |
| **2.1.1 Generic** | | | | | | | | | |
| Lorig 1999 [50] | -0.77 | 5.6 | 561 | -0.54 | 6.3 | 391 | 21.0% | -0.04 [-0.17, 0.09] | 1999 |
| Lorig 2003 [51] | -0.475 | 2.78 | 265 | -0.034 | 2.44 | 178 | 12.7% | -0.17 [-0.36, 0.02] | 2003 |
| Fu 2003 [36] | -1.01 | 9.41 | 430 | -0.84 | 7.76 | 349 | 18.9% | -0.02 [-0.16, 0.12] | 2003 |
| **Subtotal (95% CI)** | | | **1256** | | | **918** | **52.6%** | **-0.06 [-0.14, 0.03]** | |
| Heterogeneity: Tau² = 0.00; Chi² = 1.61, df = 2 (P = 0.45); I² = 0% | | | | | | | | | |
| Test for overall effect: Z = 1.32 (P = 0.19) | | | | | | | | | |
| **2.1.2 Disease specific** | | | | | | | | | |
| Lorig 1985 [59] | -0.46 | 1.98 | 129 | -0.26 | 3.07 | 61 | 5.9% | -0.08 [-0.39, 0.22] | 1985 |
| Lorig 1986 [60] | -0.3 | 4.8 | 27 | -0.7 | 2.4 | 29 | 2.2% | 0.11 [-0.42, 0.63] | 1986 |
| Lorig 1999 [58] | -0.06 | 2.9 | 189 | -0.24 | 1.7 | 97 | 8.6% | 0.07 [-0.17, 0.32] | 1999 |
| Barlow 2000 [33] | 0.09 | 1.5392 | 86 | -0.11 | 1.7298 | 78 | 5.8% | 0.12 [-0.18, 0.43] | 2000 |
| Lorig 2008 [61] | 0.028 | 3.14 | 179 | -0.064 | 2.64 | 173 | 11.0% | 0.03 [-0.18, 0.24] | 2008 |
| Lorig 2009 [41] | 0.219 | 3.88 | 161 | -0.106 | 3.37 | 133 | 9.5% | 0.09 [-0.14, 0.32] | 2009 |
| Kelly 2017 [46] | 0.14 | 0.37 | 61 | -0.02 | 0.33 | 62 | 4.4% | 0.45 [0.10, 0.81] | 2017 |
| **Subtotal (95% CI)** | | | **832** | | | **633** | **47.4%** | **0.09 [-0.02, 0.19]** | |
| Heterogeneity: Tau² = 0.00; Chi² = 5.58, df = 6 (P = 0.47); I² = 0% | | | | | | | | | |
| Test for overall effect: Z = 1.62 (P = 0.11) | | | | | | | | | |
| **Total (95% CI)** | | | **2088** | | | **1551** | **100.0%** | **0.01 [-0.07, 0.09]** | |
| Heterogeneity: Tau² = 0.00; Chi² = 11.55, df = 9 (P = 0.24); I² = 22% | | | | | | | | | |
| Test for overall effect: Z = 0.24 (P = 0.81) | | | | | | | | | |
| Test for subgroup differences: Chi² = 4.36, df = 1 (P = 0.04), I² = 77.1% | | | | | | | | | |

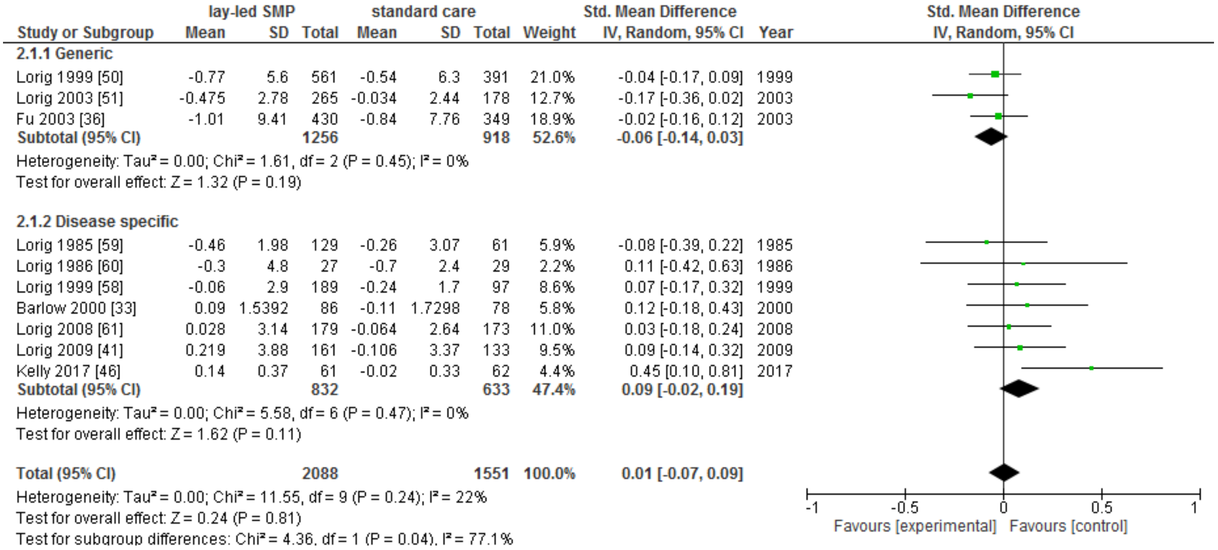


Numbers in brackets refer to reference list in the article.

## Appendix Figure 2: Number of emergency department visits, short follow-up

| | lay-led SMP | | | standard care | | | | Mean Difference | |
|---|---|---|---|---|---|---|---|---|---|
| Study or Subgroup | Mean | SD | Total | Mean | SD | Total | Weight | IV, Random, 95% CI | Year |
| **2.2.1 Generic** | | | | | | | | | |
| Lorig 1999 [50] | -0.77 | 5.6 | 561 | -0.54 | 6.3 | 391 | 0.7% | -0.23 [-1.01, 0.55] | 1999 |
| Fu 2003 [36] | -0.04 | 0.96 | 430 | -0.03 | 0.72 | 349 | 32.0% | -0.01 [-0.13, 0.11] | 2003 |
| Lorig 2003 [51] | -0.083 | 0.622 | 265 | 0.101 | 0.722 | 178 | 26.5% | -0.18 [-0.31, -0.05] | 2003 |
| **Subtotal (95% CI)** | | | **1256** | | | **918** | **59.2%** | **-0.10 [-0.24, 0.05]** | |
| Heterogeneity: Tau² = 0.01; Chi² = 3.90, df = 2 (P = 0.14); I² = 49% | | | | | | | | | |
| Test for overall effect: Z = 1.33 (P = 0.18) | | | | | | | | | |
| **2.2.2 Disease specific** | | | | | | | | | |
| Lorig 2008 [61] | -0.107 | 0.82 | 179 | -0.081 | 0.943 | 173 | 13.1% | -0.03 [-0.21, 0.16] | 2008 |
| Lorig 2009 [41] | -0.012 | 0.742 | 186 | 0.113 | 0.885 | 159 | 14.7% | -0.13 [-0.30, 0.05] | 2009 |
| Kelly 2017 [46] | -0.01 | 0.53 | 61 | 0.02 | 0.52 | 62 | 13.0% | -0.03 [-0.22, 0.16] | 2017 |
| **Subtotal (95% CI)** | | | **426** | | | **394** | **40.8%** | **-0.06 [-0.17, 0.04]** | |
| Heterogeneity: Tau² = 0.00; Chi² = 0.76, df = 2 (P = 0.68); I² = 0% | | | | | | | | | |
| Test for overall effect: Z = 1.18 (P = 0.24) | | | | | | | | | |
| **Total (95% CI)** | | | **1682** | | | **1312** | **100.0%** | **-0.08 [-0.15, -0.01]** | |
| Heterogeneity: Tau² = 0.00; Chi² = 4.82, df = 5 (P = 0.44); I² = 0% | | | | | | | | | |
| Test for overall effect: Z = 2.33 (P = 0.02) | | | | | | | | | |
| Test for subgroup differences: Chi² = 0.15, df = 1 (P = 0.70), I² = 0% | | | | | | | | | |

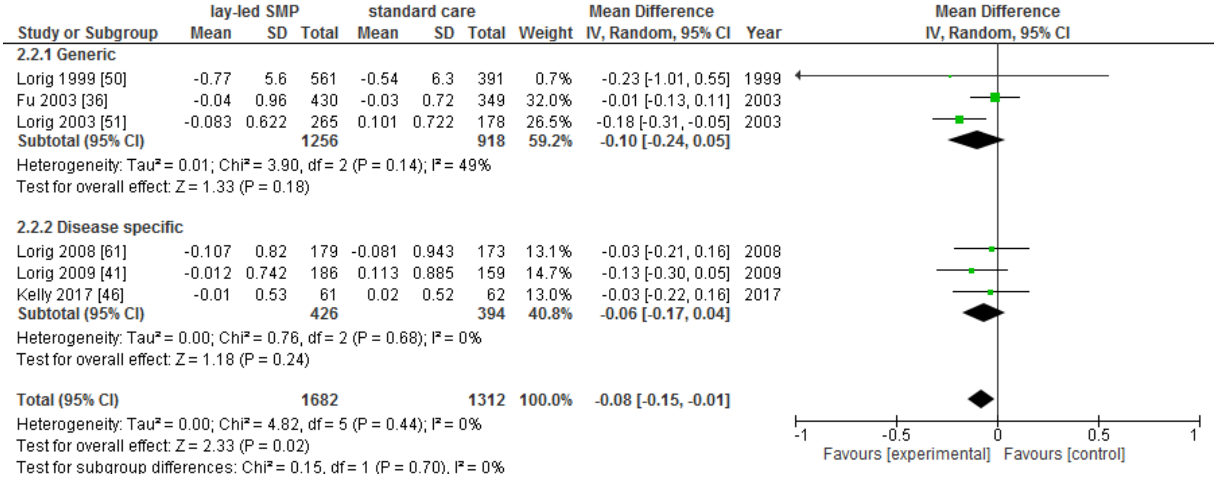


Numbers in brackets refer to reference list in the article.

## Appendix Figure 3: Number of hospital admission, short Follow-up

| | lay-led SMP | | | standard care | | | | Mean Difference | |
|---|---|---|---|---|---|---|---|---|---|
| Study or Subgroup | Mean | SD | Total | Mean | SD | Total | Weight | IV, Random, 95% CI | Year |
| Lorig 1999 [50] | -0.07 | 0.69 | 561 | -0.05 | 1.1 | 391 | 44.9% | -0.02 [-0.14, 0.10] | 1999 |
| Fu 2003 [36] | -0.06 | 0.46 | 430 | 0.06 | 0.92 | 348 | 55.1% | -0.12 [-0.23, -0.01] | 2003 |
| **Total (95% CI)** | | | **991** | | | **739** | **100.0%** | **-0.08 [-0.17, 0.02]** | |

Heterogeneity: Tau² = 0.00; Chi² = 1.46, df = 1 (P = 0.23); I² = 31%
Test for overall effect: Z = 1.51 (P = 0.13)

Mean Difference
IV, Random, 95% CI

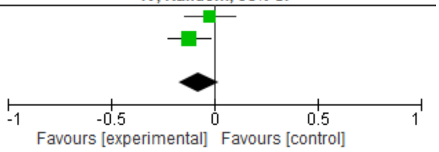


Numbers in brackets refer to reference list in the article.

## Appendix Figure 4: Length of stay in the hospital, short Follow-up

| | lay-led SMP | | | standard care | | | | Mean Difference | |
|---|---|---|---|---|---|---|---|---|---|
| Study or Subgroup | Mean | SD | Total | Mean | SD | Total | Weight | IV, Random, 95% CI | Year |
| **2.4.1 Generic** | | | | | | | | | |
| Lorig 1999 [50] | -0.28 | 5.2 | 561 | 0.56 | 7 | 391 | 19.8% | -0.84 [-1.66, -0.02] | 1999 |
| Lorig 2003 [51] | -0.011 | 0.836 | 265 | -0.079 | 3.54 | 178 | 29.5% | 0.07 [-0.46, 0.60] | 2003 |
| Fu 2003 [36] | -0.55 | 9.6 | 430 | 0.44 | 6.72 | 349 | 12.7% | -0.99 [-2.14, 0.16] | 2003 |
| **Subtotal (95% CI)** | | | **1256** | | | **918** | **62.0%** | **-0.48 [-1.20, 0.25]** | |
| Heterogeneity: Tau² = 0.24; Chi² = 4.92, df = 2 (P = 0.09); I² = 59% | | | | | | | | | |
| Test for overall effect: Z = 1.30 (P = 0.19) | | | | | | | | | |
| **2.4.2 Disease specific** | | | | | | | | | |
| Lorig 2008 [61] | 0.354 | 7.18 | 179 | -0.087 | 1.49 | 173 | 14.0% | 0.44 [-0.63, 1.52] | 2008 |
| Lorig 2009 [41] | 0 | 4.31 | 186 | -0.143 | 1.75 | 159 | 24.1% | 0.14 [-0.53, 0.82] | 2009 |
| **Subtotal (95% CI)** | | | **365** | | | **332** | **38.0%** | **0.23 [-0.35, 0.80]** | |
| Heterogeneity: Tau² = 0.00; Chi² = 0.21, df = 1 (P = 0.65); I² = 0% | | | | | | | | | |
| Test for overall effect: Z = 0.78 (P = 0.44) | | | | | | | | | |
| **Total (95% CI)** | | | **1621** | | | **1250** | **100.0%** | **-0.18 [-0.66, 0.31]** | |

Heterogeneity: Tau² = 0.13; Chi² = 7.30, df = 4 (P = 0.12); I² = 45%
Test for overall effect: Z = 0.72 (P = 0.47)
Test for subgroup differences: Chi² = 2.25, df = 1 (P = 0.13), I² = 55.5%

Mean Difference
IV, Random, 95% CI

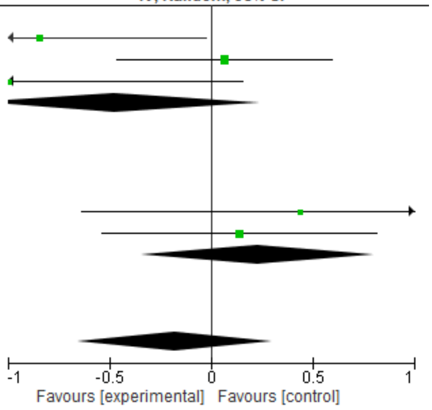


Numbers in brackets refer to reference list in the article.

**Appendix Table 1: Characteristics of included studies, n=49**

| Study* | Sample size | Outcomes | Follow-up (months) | Diseases | Intervention | Intensity (hours) | Country |
|---|---|---|---|---|---|---|---|
| **Included in meta-analysis** | | | | | | | |
| Lorig 1985 [59] | 286 | PV | 4, 8, 20 | arthritis | ASMP° | – | USA |
| Lorig 1986 [60] | 100 | PV | 4 | arthritis | ASM° | | USA |
| Lorig 1999 [50] | 1140 | PV, ED, LOS | 4 | diagnosis of chronic lung disease (asthma, chronic bronchitis, or emphysema), heart disease (coronary artery disease or congestive heart failure), stroke, chronic arthritis | CDSMP | 17.5 | USA |
| Lorig 1999 [58] | 331 | PV | 4, 12 | arthritis | SDSMP° | 12 | USA |
| Barlow 2000 [33] | 544 | PV, PV arthritis | 6 | arthritis | ASMP° | 12 | UK |
| Fu 2003 [36] | 954 | PV, ED, LOS | 6 | hypertension, heart disease (coronary heart disease or congestive heart disease), chronic lung disease (asthma, chronic bronchitis, or emphysema), arthritis, stroke, diabetes | Shanghai CDSMP | 15 | China |
| Lorig 2003 [51] | 551 | PV, ED, LOS | 6, 12 | heart disease, hypertension, diabetes, lung disease including asthma, chronic bronchitis, emphysema | Tomando | 15 | USA |
| Lorig 2008 [61] | 567 | PV, ED, LOS | 6, 18 | type 2 diabetes | SDSMP° | 15 | USA |
| Lorig 2009 [41] | 345 | PV, ED, LOS | 6, 12 | type 2 diabetes | DSMP° | 15 | USA |
| Kelly 2017 [46] | 151 | PV, ED, screening, doctor-patient relationship | 6.12 | serious mental illness | The Bridge° | – | USA |

| Study | Sample size | Outcomes | Follow-up (months) | Diseases | Intervention | Intensity (hours) | Country |
|---|---|---|---|---|---|---|---|
| **RCTs** | | | | | | | |
| de Weerdt 1991 [76] | 558 | PV, PV (GP), LOS | 6 | diabetes | EP° | 12 | NL |
| Clarke 2000 [90] | 114 | ED, LOS | 24 | serious mental illness | Peer Support° | – | USA |
| Hopman-Rock 2000 [49] | 119 | PV | 6 | arthritis | SMP for patients with osteoarthritis° | 12 | NL |
| Craig 2004 [35] | 45 | HA, LOS | 12 | serious mental illness | Peer Support° | – | UK |
| Riegel 2004 [43] | 88 | LOS, HA, Re-HA | 6 | congestive heart failure | Peer Support° | – | USA |
| Griffiths 2005 [38] | 476 | PV | 4 | diabetes, arthritis, respiratory or cardiovascular disease | CDSMP | 18 | UK |
| Haas 2005 [39] | 120 | PV, Chiro, AP, HCU | 6 | low back pain | CDSMP° | 15 | USA |
| Buszewicz 2006 [34], Patel 2009 [94] | 812 | PV, PV (home), PV (telephone), PT, Ergo, Nurse, ED, HA | 4, 12 | arthritis | SMP for arthritis° | – | UK |
| Lorig 2006 [74] | 958 | PV, HA, LOS | 12 | heart disease, chronic lung disease, type 2 diabetes | Internet-based CDSMP | 18–36 | USA |
| Swerissen 2006 [78] | 728 | PV (GP), SP, AP, Psy, ED | 6 | chronic diseases | CDSMP | 15 | AU |
| Carroll 2007 [92] | 247 | HA, LOS | 1.5, 3, 6, 12 | heart disease | Peer Support° | – | USA |
| Kennedy 2007 [40], Richardson 2008 [95], Rogers 2008 [96] | 629 | PV (GP), OUT, LOS, HCU | 6 | musculoskeletal, endocrine, circulatory, myalgic encephalitis/ chronic fatigue, respiratory, mental health, neurological | EPP | – | UK |
| Elzen 2008 [77] | 129 | PV (GP), SP, PT, LOS, HA, HCU | 6 | angina pectoris or heart failure, chronic obstructive pulmonary disease or asthma, arthritis, diabetes | CDSMP | 15 | NL |
| Lorig 2008 [52] | 855 | PV, Chiro, PT, ED, LOS | 6, 12 | osteoarthritis, rheumatoid arthritis, fibromyalgia | ASMP° | 18–36 | USA |
| Jerant 2009 [47] | 415 | LOS | 12 | arthritis, asthma, chronic obstructive pulmonary disease, congestive heart failure, depression, diabetes mellitus | Homing in on Health | 12 | USA |
| Smeulders 2009 [44] | 317 | PV (GP), Cardio, SP, ED, N, HA, LOS | 12 | congestive heart failure | CDSMP° | 15 | NL |
| Chan 2011 [75] | 772 | PV, PV (GO), ED, LOS | 6 | hypertension, heart disease, chronic lung disease, osteoarthritis, diabetes, stroke | CDSMP | 15 | China |
| Ackerman 2012 [32] | 130 | PV (GP), HP, RH, PT, HCU | 1.5, 3, 12 | arthritis | ASMP° | 15 | AU |
| Goldberg 2013 [91] | 63 | ED | 2 | serious mental illness | Living Well | 16.25 | USA |
| Adepoju 2014 [45] | 376 | ED, HA | 24 | type 2 diabetes | CDSMP | 15 | USA |
| McGowan 2015 [42] | 361 | PV, HA, LOS | 6, 12 | type 2 diabetes | CDSMP, DSMP | – | CA |
| Johansson 2017 [48] | 328 | HA, LOS | 24 | type 2 diabetes | Peer Support° | – | AT |

| Study | Sample size | Outcomes | Follow-up (months) | Diseases | Intervention | Intensity (hours) | Country |
|---|---|---|---|---|---|---|---|
| **One-group pretest-posttest** | | | | | | | |
| McGowan 2000 [85] | 64 | PV, HA, LOS, HCU | 6 | chronic diseases | CDSMP | 17.5 | CA |
| Lorig 2001 [79] | 831 | PV, ED, HA, LOS | 12, 24 | heart disease, chronic lung disease, stroke, arthritis | CDSMP | 16.8 | USA |
| McGowan 2003 [86] | 132 | PV, HP, ED, HA, LOS | 6 | chronic diseases | CDSMP | 17.5 | CA |
| Wright 2003 [89] | 185 | PV, SP, ED, LOS | 6 | chronic diseases | CDS | 12 | UK |
| McGowan 2004 [57] | 329 | PV, ED, HA, LOS | 6 | diabetes | CDSMP° | 15 | CA |
| Lorig 2005 [53] | 445 | PV, ED, LOS | 6, 12 | type 2 diabetes and other chronic diseases | Tomando | 15 | USA |
| Goeppinger 2007 [37] | 416 | PV, HA, LOS | 6, 12 | arthritis | CDSMP, ASHC° | 15 | USA |
| Gitlin 2008 [84] | 569 | PV, ED, HA, LOS, HCU | 6 | diabetes, high blood pressure, asthma, COPD, heart disease, arthritis, cancer | CDSMP | 15 | USA |
| Lorig 2008 [54] | 1074 | PV (GP), ED, P, PT, LOS | 6, 12 | type 1 diabetes, type 2 diabetes, hypertension, lung disease (like COPD, asthma and other), heart disease, arthritis, mental health condition, chronic fatigue syndrome, multiple scleroses, back problems | EPP online | 12 | UK |
| Drenkard 2012 [56] | 49 | ED, OUT, HA | 4 | systemic lupus erythematosus | CDSMP° | 15 | USA |
| Gamboa Moreno 2013 [83] | 173 | PV, ED, HA | 6 | type 2 diabetes | SDSMP° | 15 | ES |
| Lorig 2013 [55] | 114 | PV, ED | 6, 12 | type 2 diabetes | CDSMP | 15 | UK |
| Lorig 2013 [82] | 254 | PV, ED, LOS | 6, 12 | arthritis, asthma, cancer, COPD, type 1 diabetes, type 2 diabetes, heart disease, lung disease, mental health condition, others | CDSMP | 12 | AU |
| Ory 2013 [87], Ory 2013 [93], Ahn 2013 [97] | 1170 | PV, ED, HA | 6 | asthma, COPD, arthritis, cancer | CDSMP | 15 | USA |
| Jaglal 2014 [80] | 106 | PV, ED, HA | 6, 12, 18 | chronic lung disease, heart disease, stroke, arthritis | CDSMP | 15 | CA |
| Liddy 2015 [81] | 228 | PV, PV (GP), SP, ED, HA | 6, 12 | chronic diseases | CDSMP | 15 | CA |
| Slesnick 2015 [88] | 14 | PV, ED, HA, LOS | 6 | chronic hemodialysis | CDSMP | 15 | USA |

* Numbers in brackets refer to reference list in the article.

**Abbreviations: °:** disease-specific; **AP:** alternative practitioner; **ASHC:** Arthritis Self-Help Course; **ASM:** Arthritis Self-Management Course; **ASMP:** Arthritis Self-Management Program; **Cardio:** cardiologist; **CDS:** Chronic Disease Self-Management Course; **CDSMP:** Chronic Disease Self-Management Program; **Chiro:** chiropractor; COPD: chronic obstructive pulmonary disease; **DSMP:** diabetes self-management program; **ED:** emergency department visits; **EP:** Education Program; **EPP:** Expert Patients Program; **Ergo:** ergo therapist; **HA:** hospital admission; **HCU:** hospital admission; **HP:** other health professional; **LOS:** length of stay; **N:** nurse; **OUT:** outpatient visits; **P:** pharmacy; **Psy:** psychologist; **PT:** physiotherapist; **PV:** physician visits; **PV arthritis:** physician visits to discuss arthritis; **PV (GP):** general practitioner visits; **RCT:** randomized controlled trial; **Re-HA:** rehospitalisation; **RH:** rheumatologist; **SMP:** Self-Management Program; **SDSMP:** Spanish Diabetes Self-Management Program; **SP:** specialist; **Tomando:** Tomando Control de su Salud (Taking Control of Your Health)
